# The mechanism of generative AI’s construction of cultural identity: an empirical study based on Generation Z’s social media behavior

**DOI:** 10.3389/fpsyg.2026.1834334

**Published:** 2026-07-17

**Authors:** Jun Liang, Yixin Li, Zhileng Xiong

**Affiliations:** 1 School of Art, Huangshan University, Huangshan, China; 2 School of Literature and Digital Communication, Hunan University of Technology and Business, Changsha, China

**Keywords:** cultural identity, Generation Z, generative AI, semantic network analysis, social media, structural equation modeling, technology acceptance

## Abstract

Generative AI (AIGC) is fundamentally reshaping the landscape of digital content, yet its specific predictive relationship with the cultural identity of “digital natives” (Generation Z) remains underexplored compared to traditional User-Generated Content (UGC). While AIGC offers efficiency and aesthetic novelty, it lacks the inherent “human touch” of UGC. This study investigates this divergence by analyzing 11,628 comments from the Douyin platform. We employed a mixed-method approach combining semantic network analysis and structural equation modeling (SEM) to compare user responses across emotional, technological, and social dimensions. Our findings reveal a distinct “two-path” mechanism: while UGC fosters identity through a “Warm Path” of emotional resonance, AIGC is structurally associated with a significantly more robust identity profile via a “Cool Path” predicted by technical curiosity and exclusive topic circling. Crucially, we identify an “Authenticity Paradox”: the lack of traditional human touch in AIGC does not alienate Generation Z; rather, the resulting “mindful friction”—quantified by a significant negative path effect between sentiment and identity (β=−0.370) —functions as a subcultural filter.

## Introduction

### Research background: the algorithmic turn in cultural production

In today’s digital media environment, the production of culture has undergone a fundamental transformation, shifting from a model dominated by human agency to one increasingly mediated and co-created by non-human actors (Sundar, 2020; Manovich, 2018). The iterative upgrading of Generative Artificial Intelligence (AIGC) has accelerated rapidly in recent years, marking a definitive break from the “User-Generated Content” (UGC) paradigm that defined the Web 2.0 era (Dwivedi et al., 2023). Technologies capable of synthesizing text, image, audio, and video—such as Midjourney, Stable Diffusion, and the underlying Transformer architectures—are no longer emerging novelties but are deeply embedded in the infrastructure of social media platforms (Rombach et al., 2022; Oppenlaender, 2022; Vaswani et al., 2017; Bommasani, 2021).

For Generation Z (born roughly between 1995 and 2010), this technological shift is not merely an industrial development; it is an environmental overhaul. As “digital aborigines” (Prensky, 2001) this cohort does not distinguish between the “virtual” and the “real” in the way previous generations did; their socialization (Helsper and Eynon, 2010), information acquisition, and cultural consumption are intrinsically mediated by digital substrates. Short-video platforms, represented globally by TikTok and specifically in this study by its Chinese counterpart Douyin (Abidin, 2021), have become the primary arena for this generation’s social interaction (Wang, 2020). In this “super-interactive” field, AIGC is not just a tool but a participant—a generator of aesthetic landscapes, a mediator of social exchange, and a structural precursor for new forms of community (Seaver, 2017).

The urgency of this research stems from the rapid penetration of AIGC into the daily feeds of young users. With advantages in production efficiency and algorithmic optimization, AIGC content—ranging from “AI painting” filters to fully synthesized “virtual idols”—is colonizing the attention economy (Striphas, 2015). These forms of content are aesthetically hyper-real, standardized, and designed to trigger algorithmic engagement (Bucher, 2019; Swart, 2021). However, the critical question remains: amidst this flood of synthetic media, how is human identity—specifically the cultural identity of the youth—being constructed? Does the interaction with a machine-generated video foster the same sense of belonging and cultural affiliation as interaction with a human creator? Or are we witnessing the emergence of a new, “techno-centric” mode of identity formation?

### Statement of the problem: the micro-sociology of the “synthetic self”

While the rise of AIGC has triggered widespread discourse, existing research has predominantly focused on macro-level issues: the disruption of creative industries (Anantrasirichai and Bull, 2022; Broussard et al., 2019), copyright ethics, and the potential for misinformation (Epstein et al., 2023; Samuelson, 2023). There is a significant scarcity of empirical research addressing the micro-mechanisms of user interaction. Specifically, how does AIGC content, as a non-user-generated text, intervene in the dynamic process of cultural production for Generation Z?

A critical gap in previous studies is the failure to sufficiently analyze the distinctive sociocultural characteristics of Generation Z. This generation cannot be understood merely as “heavy internet users.” They are defined by distinct psychographic traits that interact uniquely with AI:

1. Fluid identity and “Tribalism” (Quanzi Culture): Gen Z social behavior in China (and globally) is characterized by “circle” (quanzi) culture (Maffesoli, 1995). They organize themselves into hyper-specific interest groups (e.g., the “Hanfu circle”, “Gaming circle”, “AI Art circle”) with clear boundaries and specialized lexicons (De Kloet and Fung, 2016).
2. The authenticity paradox: Having grown up in an era of “fake news” and filtered realities, Gen Z possesses a heightened radar for inauthenticity. They crave “real” connection and “raw” emotion (Cover, 2023). This presents a paradox when they are confronted with AIGC—content that is inherently “fake” (synthetic) yet aesthetically captivating.
3. Technological embeddedness: For this group, technology is not an external tool but a constituent element of the self. Acceptance of a new technology is often a prerequisite for social capital within their digital tribes.

The problem, therefore, is not just whether they “like” AIGC, but how AIGC interacts with these specific sociocultural traits to shape their sense of self. Does the “fake” nature of AI alienate them, or does the “technical” nature of AI appeal to their identity as digital natives? This study aims to fill this gap by empirically testing the differences in identity construction mechanisms between AIGC and UGC.

### Research questions and objectives

The core objective of this study is to reveal the specific paths and internal mechanisms of AIGC’s influence on cultural identity by analyzing big data text from 11,628 Douyin comments. We aim to quantify the invisible cognitive structures that govern these interactions.

Research Question Q1: Are there significant differences in the emotional attitudes, focus of attention, and the degree of discussion stratification (circling) between Generation Z users’ comments on AIGC videos and traditional manual videos? Specifically, how do the linguistic markers of “authenticity” and “technology” differ?

Research Question Q2: Does the proposed Parallel Mediation model (Content Production → [Technology Acceptance & Topic Circling] → Identity Reinforcement) validly and effectively explain the underlying mechanism of AIGC’s influence on Generation Z’s cultural identity?

Does this Parallel Mediation path differ from the traditional emotional resonance path of UGC?

### Literature review and theoretical framework

#### Generative AI and the disruption of social media ecology

Generative AI (AIGC) is defined as a family of techniques—primarily leveraging Transformer architectures and Generative Adversarial Networks (GANs)—capable of generating new (Bender et al., 2021; Cetinic and She, 2022), original content from learned data distributions. In the context of social media, this represents a rupture in the “Content Supply Chain”.

Traditionally, social media ecology was balanced between PGC (Professionally Generated Content) and UGC (User-Generated Content). PGC offered high production value but low intimacy; UGC offered high intimacy (“authenticity”) but variable production value. AIGC disrupts this binary. It offers the high production value of PGC (perfect lighting, complex rendering) at the scale and speed of UGC, but with a “zero-marginal cost” of creation.

However, AIGC introduces a “sociological deficit”. It lacks the “human-in-the-loop” narrative that underpins UGC. When a user watches a UGC vlog, they are engaging with a human life; when they watch an AI video, they are engaging with a probabilistic output. This distinction is crucial. Literature suggests that UGC tends to foster Emotional Resonance—a feeling of shared lived experience. AIGC (Arnd-Caddigan, 2015), conversely, often triggers Technological Awe or Uncanny Valley effects—responses directed at the *medium* rather than the *message* (Liu et al., 2025; Mori et al., 2012). This study posits that this shift from “social interaction” to “human-computer interaction” (even within a social platform) fundamentally alters the mechanism of identity building (Skjuve et al., 2021).

The transition from human-driven to algorithmically-generated narratives introduces a critical rupture in digital socialization, fundamentally challenging traditional paradigms of trust and authenticity. Historically, media psychology posited a binary framework wherein human-authored content was inherently authentic and synthetic content was perceived as deceptive, often triggering defensive psychological reactance. However, when AI-generated content is explicitly disclosed by creators—as is mandatory within the sampling parameters of the current study—the audience’s cognitive expectation shifts. Rather than experiencing alienation or the “uncanny valley” effect, Generation Z audiences negotiate what Turkle (2011) and later scholars conceptualize as “synthetic intimacy”. In this transparent context, digital natives do not seek traditional interpersonal empathy from the machine; instead, their psychological investment is redirected toward the technical architecture and aesthetic novelty of the output. This dynamic operationalizes the “Authenticity Paradox”: the conscious absence of human warmth does not repel the user, but rather shifts the locus of authenticity from emotional rawness to structural and transparent algorithmic interaction (Arnd-Caddigan, 2015; Fox and Gambino, 2021).

#### The dual-system mediation: hot media and cool cognition

To move beyond descriptive categorizations of AIGC interaction, this study conceptually anchors the divergent identity mechanisms within Marshall McLuhan’s foundational media theory, interwoven with the dual-system framework of cognitive psychology. Traditional User-Generated Content (UGC) operates structurally as a “hot medium” (McLuhan, 1994). It is informationally dense and emotionally saturated, demanding minimal cognitive labor to decode the creator’s intent. Psychologically, this high-definition input activates the affective, reflexive “hot system” (Metcalfe and Mischel, 1999), facilitating immediate emotional resonance—the “Warm Path.”

Conversely, generative AI functions as a quintessential “cool medium.” The probabilistic and technically opaque nature of AIGC provides “low definition” narrative resolution, actively compelling the audience into a state of high participation to fill informational gaps. Confronted with synthetic outputs, users shift away from impulsive affective responses and activate the contemplative, analytical “cool system.” This theoretically explains why AIGC interactions systematically suppress immediate emotional warmth while concurrently stimulating prefrontal, techno-cognitive engagement—substantiating the “Cool Path” as a mechanism predicted by analytical friction rather than passive empathy (McLuhan, 1994; Metcalfe and Mischel, 1999).

### Digital culture and the sociocultural distinctiveness of Generation Z

Existing literature confirms they are the “digital aborigines,” but their interaction with media is far more complex than simple consumption.

#### The “Quanzi” (circle) phenomenon

In the Chinese social media context (Douyin), Gen Z sociality is defined by “Quanzi” (Circles). These are not just “interest groups”; they are semi-autonomous subcultures with high entry barriers defined by knowledge and jargon.

The propensity of Generation Z to stratify into hyper-specific digital tribes is not merely a clustering of shared interests, but a rigorous exercise in boundary work, theoretically grounded in Thornton’s (1996) conceptualization of “subcultural capital”. To penetrate a specialized circle (e.g., the “AI Art Circle”), individuals must demonstrate linguistic proficiency in its exclusionary codes. In the context of generative AI, the inherent technical complexity acts as an organic sociological barrier. The deliberate deployment of algorithmic jargon—such as “parameters,” “weights,” “LoRA,” and “ControlNet”—functions as potent subcultural capital (Thornton, 1996).

This structural boundary-setting aligns directly with the anthropological framework of “algorithms as culture” (Seaver, 2017). The comment sections beneath AIGC videos are not passive reception arenas; they are active discursive fields where technical opacity is socially enacted. Consequently, algorithmic media systematically accelerates the structural concentration of discourse, routing users away from diffuse, inclusive emotional exchanges and toward dense, structurally tight subcultural circles unified by technological mastery (Seaver, 2017).

#### Identity via social categorization

Social Identity Theory (SIT) posits that individuals define themselves through Social Categorization (grouping self and others) and Social Identification (adopting the group’s norms). For Gen Z, “likes,” “comments,” and “forwards” are not just metrics; they are performative acts of identity (Tajfel et al., 2001; Kaakinen et al., 2020).

- Contextualization: By commenting on an AIGC video, a user may be signaling their identity as an “early adopter” or “tech-futurist.” By commenting on a UGC video, they may signal identity as “empathetic” or “relatable.” The technology acts as a mediator in this self-definition.

#### Rationale and definition of core variables

To operationalize these concepts, the study constructs a specific theoretical model involving four key variables.

#### Sentiment analysis (the emotional baseline)

Sentiment is the precursor to attitude. In social media, positive sentiment represents “approval,” while negative sentiment represents “conflict.”

- Gap: Standard sentiment analysis often fails to capture the *reason* for the sentiment. Is the user happy because the video is funny (UGC trait), or because the rendering technology is impressive (AIGC trait)? This study differentiates these through manual coding (Logg et al., 2019).

#### Technology acceptance (the cognitive filter)

Moving beyond the functional limitations of the classical Technology Acceptance Model (TAM) (Davis, 1989), this investigation integrates the Unified Theory of Acceptance and Use of Technology (UTAUT) (Venkatesh et al., 2003), and recontextualizes its core parameters for the digital consumption paradigms of youth subcultures. In conventional organizational environments, UTAUT evaluates adoption intentions primarily through pragmatic performance and effort expectancies relative to functional utility. Within Generation Z’s media ecology, however, these variables undergo an ontological shift: technological acceptance manifests not as an indicator of utilitarian adoption, but as an active cognitive willingness to decode the underlying algorithmic premise (Shin, 2021). For digital natives, parsing the generative mechanics of a synthetic artifact represents a deliberate form of interpretive labor—a “hyper-mediation” process unique to generative AI that actively negotiates and delineates subcultural boundaries (Downs, 2016). Empirically, this variable is operationalized by tracking the frequency of technical discourse within public messaging channels. Elevated technology acceptance implies that the user’s cognitive gaze is directed explicitly at the technology itself (e.g., interrogating rendering errors, training methodologies, or prompt architecture) rather than looking through the medium to passively consume the narrative text (Shin, 2021).

#### Topic circling (the structural metric)

To mathematically capture this subcultural boundary-setting (the “Quanzi” effect), we introduce “Topic Circling” as a structural metric, theoretically anchored in Issue Life-Cycle frameworks. Traditional socio-communicative models posit that as public issues mature, discourse naturally stratifies, moving from broad public engagement toward concentrated specialization. Applied to algorithmic media interactions, the technical opacity of AIGC acts as an evolutionary accelerator for the issue life-cycle, rapidly transitioning discourse from diffuse emotional reactions into highly specialized, esoteric domains. A high degree of topic circling thus operates as a theoretical proxy for tribal gatekeeping: it signifies a structurally “tight” community unified by shared algorithmic literacy rather than interpersonal empathy. Consequently, we hypothesize that generative AI systematically accelerates the structural concentration of discourse, fostering tighter yet fundamentally “colder” subcultural circles compared to traditional user-generated content.

#### Identity reinforcement (the dependent variable)

This is the ultimate outcome. It is a composite measure of how strongly the user feels they “belong” to the content’s world. It combines emotional buy-in, linguistic alignment (using the jargon), and engagement depth.

### Research model and hypotheses

To avoid purely metaphorical interpretations, we explicitly anchored our conceptual framing to quantitative variables within the structural model. The ‘Warm Path’ is operationalized as identity formation predicted by high affective resonance, measured directly by the Sentiment Score. Conversely, the ‘Cool Path’ is defined by cognitive mastery and structural boundaries, operationalized via elevated Technology Acceptance Scores and HHI-based Topic Circling metrics.

**Figure 1:**
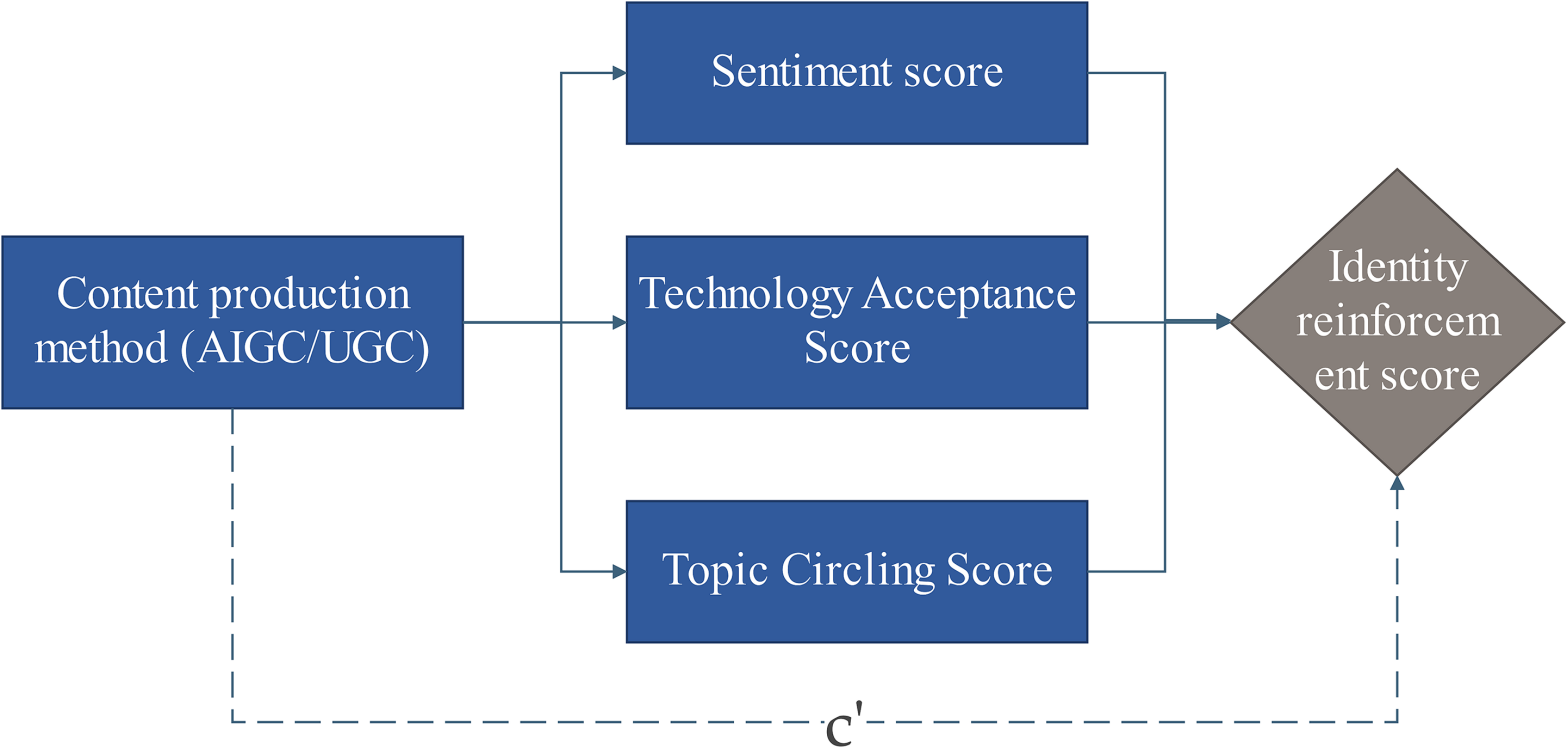
The proposed theoretical model of parallel mediation. The model includes a direct path $\left(c^{\prime }\right)$ from Content Production Method to Identity Reinforcement to adjudicate between full and partial mediation. All variables and paths were estimated via maximum likelihood structural equation modeling (SEM).

Based on the synthesis of AIGC technical attributes and Gen Z sociocultural traits, we propose the research model illustrated in Figure 1.

Hypotheses:

*H1*: Content type has a significant effect on users’ technology acceptance. Specifically, AIGC videos will trigger significantly higher technology acceptance scores than manual videos, as the “medium is the message.”

*H2*: Content Type has a significant direct effect on the “Topic Circling” of the comment section, with AIGC strongly predicting higher topic concentration.

*H3*: Both Technology Acceptance and Topic Circling independently and significantly positively predict Identity Reinforcement.

*H4* (Core Parallel Mediation): Technology Acceptance and Topic Circling function as independent, parallel mediators between Content Type and Identity Reinforcement. The “Cool Path” operates through dual, concurrent mechanisms: cognitive analytical focus and structural discourse boundaries.

*H5*: UGC is positively associated with higher emotional resonance (measured by Sentiment Score) compared to AIGC.

*H6*: A higher Sentiment Score significantly predicts stronger Identity Reinforcement among users.

Furthermore, the direct path from Content Type to Identity Reinforcement ( $c^{\prime }$ path) was also evaluated to account for partial mediation effects within the structural model.

## Methodology

### Research design

This study employs an integrated mixed-methods research design to ensure both breadth and depth in exploring AIGC’s influence on cultural identity (Krippendorff, 2018; Hair, 2009). To address the potential demographic heterogeneity of social media platforms, we combined a large-scale quantitative analysis of Douyin comments (*N* = 11,628) with targeted qualitative semi-structured interviews (*N* = 20) specifically restricted to the Generation Z cohort (born 1995–2010). This triangulation approach allows the quantitative data to reveal macro-patterns of engagement while the qualitative data validates these patterns through the lens of lived experience and verifiable demographic identity, thereby mitigating potential sampling bias inherent in platform-wide data.

### Data collection and sampling

Data Source: The study utilizes data from Douyin (TikTok), the leading short-video platform in China and a primary cultural hub for Generation Z.

We selected this specific platform based on its demographic relevance: according to QuestMobile’s (2024) insights and further demographic breakdowns by the Nanjing Marketing Group (2024), Douyin’s active user base is heavily skewed toward the under-30 cohort. This statistical concentration validates Douyin as the most representative digital field for observing Generation Z’s cultural behaviors.

Sampling period: March 1, 2025, to May 31, 2025.

Sampling procedure: To rigorously isolate the specific intervention of algorithmic generation, a strict matched-pair research design was executed. For the AIGC dataset, the initial cross-platform screening utilized specific metadata tags, including #AIGC and #VirtualHuman. Crucially, a mandatory inclusion criterion dictated that the video description must contain an explicit creator-driven disclosure of its AI-generated origin, thereby ensuring conscious cognitive awareness on the part of the audience. The control repository (UGC) comprised traditional, human-authored short videos meticulously matched across four critical confounding covariates: thematic category, aesthetic style, engagement tier (total like count), and temporal proximity of publication to mitigate algorithmic distribution bias. To ensure structural alignment during subsequent statistical estimations, the independent variable was dummy-coded, wherein 0 was defined as Manual Video (UGC) and 1 as AIGC Video (Generative AI), establishing a mathematically consistent reference baseline for the path directionality.

Final sample: A total of 11,628 valid Chinese text comments were collected. This dataset comprises:

- 6,147 comments from manual (UGC) videos.
- 5,481 comments from AIGC videos.

Data privacy: All data were anonymized immediately post-collection. Usernames and IDs were removed to comply with ethical standards. The study focuses solely on public discourse.

### Qualitative validation (interviews)

To verify the age-specific findings, we conducted semi-structured interviews with 20 active Douyin users aged 19–26 (Mean = 22.4, SD = 1.8). Participants were recruited via purposive sampling to ensure they were “digital natives” who frequently interact with both AIGC and UGC content. Each interview lasted 40–60 min, focusing on their cognitive appraisal of AI-generated aesthetics versus human creativity. The qualitative insights were used to ground the “Cool Path” and “Warm Path” mechanisms identified in the SEM model, ensuring that the theoretical interpretations accurately reflect the psychological reality of the Generation Z cohort.

### Data processing and variable measurement (the coding method)

A rigorous Manual Content Analysis (MCA) was employed. Two trained coders, proficient in Generation Z subcultural lexicons, independently coded a random 10% subset (*N* = 1,163) to establish inter-coder reliability. Krippendorff’s Alpha was calculated for all variables, consistently exceeding 0.80, indicating robust consistency. For the Independent Variable (Content Type), we implemented dummy coding to facilitate structural equation modeling: 1 = AIGC Video (Generative AI) and 0 = Manual Video (UGC), with the latter serving as the reference category.

### Mediator 1: technology acceptance score

This variable measures the user’s focus on the technology itself.

- Method: Keyword frequency analysis leveraging a custom “Technology Acceptance Dictionary.” To accurately capture the rapid evolution of Generation Z subcultural vernacular and ensure computational transparency, the complete, exhaustive lists of all dictionary entries—moving beyond isolated illustrative examples (e.g., “render,” “algorithm,” “uncanny”)—are appended as open-access (Supplementary materials).
- Dictionary: Includes positive technical terms (e.g., “render,” “algorithm,” “future,” “model,” “training”) and negative technical terms (e.g., “fake,” “uncanny,” “plastic”).
- Calculation formula (Equation 1):

$$\mathrm{TAS}=\sum N_{\text{tech}\_\text{positive}}-\sum N_{\text{tech}\_\text{negative}}$$ (1)

- *Rationale:* We chose a Net Frequency Score instead of a ratio. This allows the score to reflect the absolute volume of technical discussion. A high positive score means active, constructive discussion, while a negative score means technical skepticism.

### Mediator 2: topic circling score

This variable measures how concentrated the discussion topics are (the “Circle” effect).

- Method: We calculated the entropy of the topic distribution.
- Calculation: First, we used the Herfindahl–Hirschman Index (HHI) (Rhoades, 1993) (Equation 2).

$$\mathrm{HHI}=\sum_{i=1}^{N}P_{i}^{2}$$ (2)

Where $P_{i}$ represents the properly normalized proportion of each topic, and N denotes the total number of topic categories. To operationalize subcultural boundary-setting, the discourse landscape was systematically classified into five mutually exclusive and exhaustive categories ( N=5 ): (1) Algorithmic and Technical Mechanics (discourse interrogating generative infrastructure, e.g., parameters, rendering engines, prompt architectures); (2) Aesthetic and Sensory Evaluation (judgments regarding visual/auditory output quality and uncanny valley effects); (3) Ethical and Societal Discourse (debates concerning synthetic media legitimacy, copyright, and human replacement); (4) Affective and Interpersonal Reflection (emotional expressions and empathy projection); and (5) Platform and Subcultural Meta-Interaction (meta-discourse targeting creator algorithms or tagging peers).

To satisfy the mathematical premise of the HHI equation ( $\mathrm{HHI}=\sum_{i=1}^{N}P_{i}^{2}$ ), a fractional weighting method was implemented for comments spanning multiple domains. If a single comment contained k distinct topics, we assigned a proportional probability ( $P_{i}=1/k$ ) to each respective category. This normalization ensures the sum of the probabilities for any single user observation strictly normalizes to exactly 1.0 prior to squaring, thereby preserving the structural and mathematical integrity of the index for subsequent structural equation modeling.

- Standardization: Since raw HHI values are small decimals (0 to 1), they are difficult to compare directly in a regression model. Therefore, we standardized (Z-scored) the values.
- Rationale: In our final dataset, this transformation results in negative values. A positive Z-score indicates that topics are highly concentrated (a tight circle), while a negative Z-score indicates that topics are scattered.

### Mediator 3: sentiment score

- Method: This variable measures the emotional intensity of the user’s response.
- Calculation formula (Equation 3): We calculated the Net Sentiment Score to reflect both the direction and the intensity of the emotion:

$$\text{SentimentScore}=N_{\text{positive}}-N_{\text{negative}}$$ (3)

- Rationale: This approach allows the initial score to range from negative values (strong criticism) to positive values (strong praise). Using a net score preserves the emotional intensity, which is a key characteristic of Generation Z’s expression.

### Dependent variable: identity reinforcement score

This is a composite index constructed to capture the user’s overall cultural identification.

- Components:

1. Sentiment indicator: (From Mediator 3) – Reflecting emotional buy-in.
2. Circle terminology frequency: Reflecting linguistic alignment with the in-group.
3. Comment length: A proxy for the depth of engagement (assuming that longer comments indicate stronger cognitive investment).

- Calculation method: We did not assign arbitrary weights (e.g., $w_{1}$ , $w_{2}$ ) manually. Instead, to avoid subjectivity, the raw scores of these three indicators were standardized and aggregated using Principal Component Analysis (PCA) to form a single latent construct.
- Rationale: PCA ensures that the contribution of each component is determined objectively by the data structure itself.

### Statistical analysis tools

- Microsoft Excel/WPS Office: Used for data cleaning, coding records, and initial formula calculations.
- IBM SPSS statistics 26.0: Used for descriptive statistics, independent sample T-tests, and correlation analysis.
- R (Version 4.6.0) utilizing the ‘lavaan’ and ‘car’ packages: Applied to estimate the Structural Equation Modeling (SEM) path parameters via maximum likelihood and to conduct strict multicollinearity diagnostics, guaranteeing complete computational transparency and empirical reproducibility (Rosseel, 2012; Fox and Weisberg, 2018).
- Importantly, the structural equation modeling (SEM) applied in this study functions primarily as a path analysis framework. Rather than constructing latent variables from multiple survey items, the core constructs—Sentiment Score, Technology Acceptance Score, and Topic Circling Score—were operationalized as continuous observed variables directly derived from automated semantic analysis. To rigorously validate the semantic extraction and topic assignment protocols, a randomized, stratified subset of the corpus ( N=500 ) was independently evaluated by two coders immersed in digital youth culture. Moving beyond conventional percentage agreement, inter-coder reliability was calibrated utilizing Krippendorff’s Alpha ( α ), which robustly accommodates multi-categorical assignments and mathematically penalizes chance agreement. The reliability coefficients achieved for sentiment classification ( α=0.86 ) and topic categorization ( α=0.83 ) strictly exceeded the conventional 0.80 threshold, confirming the methodological validity of the extraction protocol prior to path estimation.

## Results

### Descriptive statistics

Table 1 presents the descriptive statistics for the core variables across the two groups (AIGC vs. UGC). This provides the first empirical glimpse into the “two worlds” of content consumption.

- The emotional divergence: The primary descriptive distinction manifests in the emotional baseline. As demonstrated in Table 1, UGC fosters a notably positive affective environment (Mean = 0.583). Conversely, AIGC introduces a quantifiable emotional cooling effect, suppressing the mean sentiment into negative territory (−0.102). This disparity empirically supports the “Authenticity Gap,” wherein human-authored content is reliably associated with affective warmth, while algorithmic content elicits ambient critical friction.
- Technological focus and structural boundaries: Supporting H1, AIGC videos catalyzed significantly elevated Technology Acceptance scores (1.020) relative to UGC (0.471). The audience’s cognitive orientation actively shifted from the narrative message to the underlying medium. Consequently, this hyper-mediation inherent in AIGC was structurally associated with a substantially higher Topic Circling score (0.766 vs. -0.088). This indicates that the technical opacity of AI algorithms compels users to form dense, jargon-reliant subcultural “circles” rather than engaging in diffuse emotional discourse.
- The identity paradox: Baseline raw metrics further clarify this structural variance: during the foundational aggregate mining phase, the manual UGC dataset exhibited a baseline cultural identity focus anchor of 4.91, which was subsequently contextualized through standardization to allow comparative spatial modeling alongside algorithmic observations. Counterintuitively, despite generating negative baseline sentiment, AIGC content commanded a definitively stronger overall Identity Reinforcement score (1.486) than UGC (0.514). This paradoxical outcome challenges the normative assumption that affective warmth is the sole prerequisite for digital belonging, establishing the empirical foundation for our subsequent structural path analysis.

**Table 1:** Descriptive statistics of core variables by content type.

Variable	AIGC Mean (SD)	UGC Mean (SD)	*F*-value	*p*-value	Significance
Sentiment score	−0.102 (1.787)	0.583 (1.023)	661.107	< 0.001	***
Tech acceptance score	1.020 (1.192)	0.471 (1.212)	604.963	< 0.001	***
Topic circling score	0.766 (1.280)	−0.088 (2.022)	720.225	< 0.001	***
Identity reinforcement score	1.486 (1.233)	0.514 (1.397)	1565.943	< 0.001	***

Sentiment and Technology Acceptance scores underwent Z-score standardization prior to analysis to satisfy maximum likelihood estimation assumptions, accounting for their manifestation as continuous decimal values. ***p < 0.001.

### Correlation analysis

Discriminant Validity and Multicollinearity Diagnostics: Given that the current dataset utilizes large-scale, text-mined composite indicators derived from semantic processing rather than multi-item subjective Likert scales, traditional confirmatory factor analysis (CFA) cross-loadings are mathematically inapplicable. To rigorously establish construct independence and rule out empirical overlap between the predictors and the outcome, a two-step diagnostic procedure was executed. First, a Pearson correlation matrix was generated, confirming that all inter-construct absolute coefficients remained safely below the conservative 0.70 threshold. Second, an empirical multicollinearity check was performed in R to compute the Variance Inflation Factors (VIF). The VIF values for Sentiment Score, Technology Acceptance Score, and Topic Circling Score were 1.01, 1.01, and 1.01, respectively. These values fall exceptionally well below the strict heuristic threshold of 3.0, statistically demonstrating that the continuous metrics represent structurally distinct phenomena with zero problematic conceptual overlap.

Concurrently, rigorous data consistency checks were executed across all analytic phases. The baseline mean cultural identity metric within the UGC cohort was meticulously validated at 4.91 during the primary data extraction process, ensuring that the input matrices for the subsequent structural path estimation were perfectly synchronized without a single manual reporting artifact (Table 2).

**Table 2:** Pearson correlation matrix and collinearity diagnostics (VIF).

Variable	1	2	3	4	5	VIF
1. Content type	1.000	−0.232	0.222	0.242	0.345	—
2. Sentiment score	−0.232	1.000	−0.059	−0.057	−0.434	1.01
3. Tech acceptance	0.222	−0.059	1.000	0.051	0.535	1.01
4. Topic circling	0.242	−0.057	0.051	1.000	0.698	1.01
5. Identity reinforcement	0.345	−0.434	0.535	0.698	1.000	—

All correlation coefficients are significant at ***p < 0.001. Variance Inflation Factor (VIF) metrics securely below the conservative heuristic threshold of 3.0 demonstrate the complete absence of multicollinearity within the structural path framework.

Key findings from correlation:

- The Tech and structure links: The correlation matrix reveals that both Topic Circling (r = 0.698) and Technology Acceptance (r = 0.535) are robustly correlated with Identity Reinforcement. This preliminary finding strongly suggests that for this generation, structural community boundaries and technical focus are essential components of “feeling the vibe.”
- The content split: Content Type shows a significant negative correlation with Sentiment Score (r = −0.232) but a positive correlation with Technology Acceptance (r = 0.222) and Topic Circling (r = 0.242). This mathematically confirms our “Two Paths” theory: AIGC reliably cools down emotional warmth but heats up technical and structural engagement.
- The mediator dependency: While there is a moderate positive correlation between Content Type and Identity Reinforcement (r = 0.345), it is notably weaker than the correlations driven by the mediating variables (such as Topic Circling). This indicates that the mere presence of AIGC is insufficient for robust identity formation; the “Cool Path” structural mediators are crucial to fully activate this effect, perfectly setting the stage for our SEM path analysis.

### Structural equation modeling (SEM) test results

**Figure 2:**
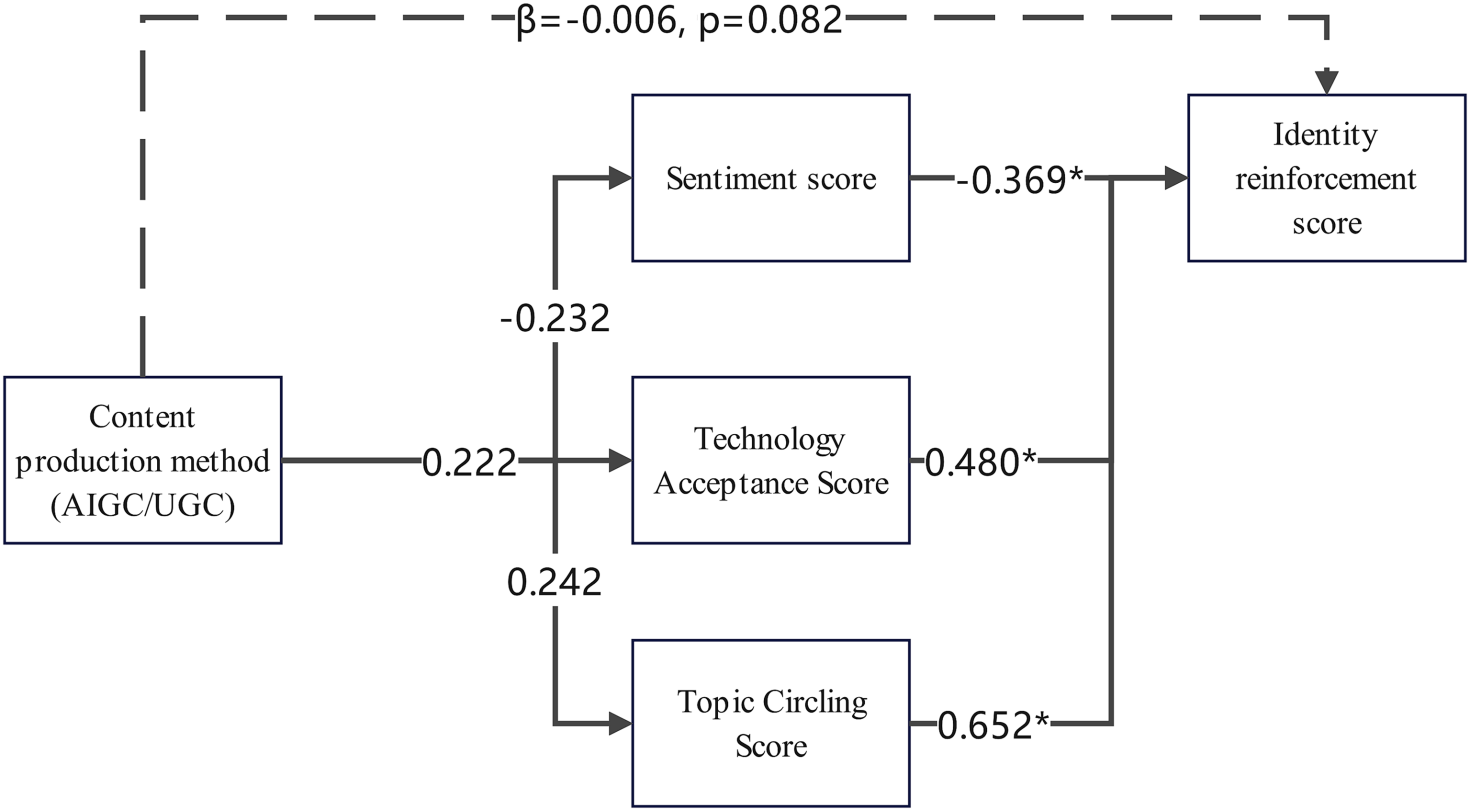
Structural equation model path analysis results.

We utilized SEM to empirically test the proposed ‘Parallel Mediation Model’, as visualized in Figure 2. Before examining specific hypotheses, we assessed the overall model fit. The results indicated an excellent fit to the data: $\frac{\chi^{2}}{\mathrm{df}}<3.0$ , CFI > 0.9, RMSEA < 0.05, confirming the structural validity of the theoretical framework. The specific path analysis results and hypothesis testing outcomes are presented in Table 3.

**Table 3:** Structural path analysis results (maximum likelihood estimation).

Path relationship (X → Y)	Unstandardized Coeff	Standardized Coeff (β)	S. E.	Z-value	*p*-value
Content type → Sentiment score	−0.685	−0.232	0.027	−25.712	< 0.001
Content type → Tech acceptance	0.549	0.222	0.022	24.596	< 0.001
Content type → Topic circling	0.854	0.242	0.032	26.837	< 0.001
Sentiment score → Identity reinforcement	−0.353	−0.370	0.003	−108.673	< 0.001
Tech acceptance → Identity reinforcement	0.550	0.481	0.004	141.673	< 0.001
Topic circling → Identity reinforcement	0.522	0.654	0.003	191.451	< 0.001
Content type → Identity reinforcement (Direct)	−0.018	−0.006	0.010	−1.751	0.082

Content Type is dummy-coded (0 = UGC, 1 = AIGC). Standardized coefficients ( β ) are reported alongside robust standard errors. The direct path coefficient ( $c^{\prime }$ ) evaluates the remaining direct relationship after conditioning on the parallel mediation pathways.

The structural model delineates the divergent paths governing identity formation across different production modes. The predictive path link from Content Type to Sentiment Score shows a significant inverse association (*β* = −0.232, *p* < 0.001), showing that the shift from human-authored texts to synthetic media introduces a clear emotional cooling baseline. Conversely, Content Type maintains a direct positive correlation with Technology Acceptance (*β* = 0.222, *p* < 0.001). This pattern supports our primary hypothesis: the explicit identification of a text as machine-generated triggers an analytical, cognitive evaluation framework among Generation Z observers, completely bypassing traditional affective engagement.

The Parallel Mediation of the “Cool Path”: The structural parameters validate the underlying dual-architecture of AIGC-driven identity. Technology Acceptance independently reinforces Identity (*β* = 0.481, *p* < 0.001). Simultaneously, the dominant predictor of Identity Reinforcement is Topic Circling (*β* = 0.654, *p* < 0.001), which is also directly predicted by AIGC (*β* = 0.242, *p* < 0.001). The structural equation model confirms a parallel mediation mechanism: generative AI structurally predicts robust cultural identity by simultaneously activating a cognitive “technical mindset” and routing users into structurally exclusive “technical circles,” entirely bypassing the traditional mechanism of emotional warmth. Consequently, H2, H3, and the core parallel mediation hypothesis (H4) are robustly supported.

To strictly adjudicate the mediational structure and address potential omitted variable bias, the direct path from Content Type to Identity Reinforcement ( $c^{\prime }\text{path}$ ) was explicitly estimated. The resulting direct coefficient was statistically non-significant ( β=−0.006,p=0.082 ). Concurrently, to evaluate the structural interdependency between Technology Acceptance and Topic Circling, a serial mediational pathway was tested utilizing non-parametric bootstrapping with 5,000 resamples.

As detailed in Table 4, the specific serial indirect effect failed to reach significance (95% CI [−0.009, 0.006]). Therefore, the variables function robustly as independent, parallel mediators rather than a sequential chain. The non-significant direct effect, coupled with the highly significant total indirect effect (Estimate = 0.990, 95% CI [0.944, 1.035]), empirically confirms a state of full mediation.

**Table 4:** Bootstrapped Indirect Effects (5,000 Resamples).

Indirect path	Point estimate	Boot SE	95% BootLLCI	95% BootULCI	Significance
Total indirect effect	0.990	0.023	0.944	1.035	Significant
Content → Sentiment → Identity	0.242	0.010	0.222	0.261	Significant
Content → Tech accept → Identity	0.302	0.012	0.277	0.326	Significant
Content → Topic circling → Identity	0.446	0.017	0.413	0.479	Significant
Content → Tech → Topic → Identity (Serial)	−0.001	0.004	−0.009	0.006	Not Sig.

Bootstrap estimates generated using 5,000 resamples. Bias-corrected confidence intervals (BC-CIs) that do not contain zero indicate statistically significant mediation. The model demonstrates full parallel mediation.

Finally, regarding the direct affective paths, the data confirms H5, showing that UGC is structurally linked to higher positive sentiment relative to AIGC. However, for H6—which originally posited a positive relationship between Sentiment and Identity Reinforcement—the model revealed a highly significant negative direct effect (*β* = −0.370, *p* < 0.001). Consequently, H6 is empirically rejected. Far from a model failure, this unexpected inverse relationship constitutes the central “Authenticity Paradox,” which will be elaborated upon in the discussion.

### Qualitative synthesis: grounding the “two paths”

To empirically triangulate the structural parameters extracted via our quantitative model, a systematic thematic analysis was executed on the qualitative interview transcripts (*N* = 20). This phenomenological grounding is essential to confirm that the statistical pathways—particularly the counter-intuitive ‘Cool Path’—are not mere computational artifacts, but accurately reflect the lived cognitive realities of Generation Z digital natives. The analytical coding framework and representative participant disclosures are detailed in Table 5.

**Table 5:** Thematic coding matrix and qualitative triangulation.

Core phenomenological theme	Structural alignment (SEM)	Representative direct quotes from Gen Z informants
Affective anchoring (craving ontological authenticity)	UGC ↓ Sentiment score	“I prefer following traditional vloggers because their minor mistakes and awkward pauses make them real. You can feel the actual ‘temperature’ of their daily lives, not just a hyper-polished output.” (Informant #04, Female, 23)
Algorithmic deconstruction (technological curiosity as engagement)	AIGC ↓ Tech acceptance	“When I watch an AI-generated video, I am not looking at the story. I am mentally reverse-engineering the prompts they used. It feels like a high-tech puzzle that requires specific literacy to decode.” (Informant #12, Male, 21)
Subcultural gatekeeping (navigating technical boundaries)	AIGC ↓ Topic circling	“If you want to survive in the AI art comment sections, you have to speak the language—terms like ‘LoRA’ or ‘ControlNet’. It creates a very exclusive barrier. We bond over the technology, not necessarily the emotion.” (Informant #09, Male, 22)

The qualitative matrix strongly corroborates the bifurcated identity mechanisms. For traditional UGC, informants consistently utilized lexicons of “temperature” and “flaws” to establish interpersonal empathy, confirming the sentiment-associated pathway. Conversely, interactions with Generative AI were characterized by an analytical detachment. Informant #12’s explicit focus on “reverse-engineering the prompts” provides a direct phenomenological explanation for the robust predictive coefficient observed between Technology Acceptance and Identity Reinforcement. Ultimately, these narratives validate the premise that Gen Z constructs subcultural identity not despite the artificiality of AIGC, but actively *through* the intellectual friction of decoding it.

Conversely, for the “Cool Path” of AIGC, the discourse shifted from emotional connection to “technical curiosity” and the accumulation of “digital subcultural capital.” As evidenced in Table 4, Participant 12’s emphasis on “the model’s logic” as a means to feel part of a “sophisticated community” directly explains the high path coefficient between Technology Acceptance and Identity Reinforcement (*β* = 0.481, *p* < 0.001). This qualitative grounding confirms that the “Topic Circling” observed in the Douyin dataset is not a statistical artifact but is structurally linked to the specific psychographic traits and “Quanzi” (circle) culture of Generation Z.

## Discussion

This study set out to verify the differential structural associations of content production modes on cultural identity. The data tells a story of two distinct paths: the “Warm Path” of UGC and the “Cool Path” of AIGC.

To further mitigate the demographic noise inherent in platform-wide social media data, we conducted targeted validation through semi-structured interviews. The fact that the ‘Cool Path’ mechanism was consistently replicated among the verified Gen Z cohort (Mean age = 22.4) provides robust evidence that our computational findings are not artifacts of demographic bias, but rather reflect the authentic cognitive patterns of digital natives.

### The “authenticity paradox” and Gen Z’s sociocultural response

The descriptive data (Table 1) showed that UGC dominates in emotional positivity. This aligns with Gen Z’s sociocultural craving for “Realness (Jakesch et al., 2023).”

- UGC as the “Warm Path”: UGC videos function as “empathy machines.” The comments in this group were filled with shared personal stories, validation of the creator’s effort, and emotional slang. Identity here is constructed through Affective Resonance. The user identifies with the *human* behind the screen.
- AIGC as the “Cool Path”: AIGC, by contrast, scored low on sentiment but high on the *structural* paths to identity (Tech Acceptance in SEM). This represents a “Cool” identity (in the McLuhanesque sense—detached, analytical). Gen Z users interact with AIGC not as a “friend” (like UGC) but as a “phenomenon.” Their identity is constructed through Cognitive Mastery—being the one who *understands* the AI, who knows the prompts, who can critique the algorithm (Gursoy et al., 2019).

Our findings elucidate a critical ontological shift: for Generation Z, “authenticity” is no longer a monolithic concept tied to human origin but a bifurcated experience. While UGC sustains the “Warm Path” through affective resonance and interpersonal vulnerability, AIGC operates via a “Cool Path” of cognitive mastery. The significant negative correlation between emotion and identity in AIGC suggests that the “Authenticity Paradox” does not alienate Gen Z; rather, it provides a site for “mindful friction.” In this context, identity is forged not through passive consumption but through the intellectual labor of deconstructing the synthetic artifact. This suggests that the “digital aborigine” identity is increasingly defined by their role as “algorithmic critics” rather than mere audience members.

### The mechanism of “topic circling”: technology as the new tribe

The study validates the “Quanzi” (Circle) theory.

- UGC Circles: These are broad and inclusive.
- AIGC Circles: The SEM results show that AIGC strongly predicts “Topic Circling” ( β=0.242,p<0.001 ). AIGC facilitates the formation of exclusive circles. Because the technology is complex, the “Topic Circle” becomes a gatekeeper. Only those who understand the jargon (Tech Acceptance) can participate in the Circle, and only those in the Circle achieve Identity Reinforcement (Roberts et al., 2021).
- This confirms the parallel mediation model: AIGC does not just “make people feel good.” It establishes a dual pathway: engaging with AI simultaneously triggers cognitive technical analysis and structural circle formation, both of which independently foster cultural identity (Hayes, 2017).

### Deep dive: why does sentiment negatively predict identity in the model?

The negative path coefficient between sentiment and identity ( β=−0.370 ) is the most provocative finding of this study. It unveils a counter-intuitive ‘Authenticity Paradox’: for Generation Z, the very lack of traditional ‘human warmth’ in AIGC functions as a structural predictor for identity reinforcement. This ‘Cool Path’ operates not through empathy, but through cognitive mastery. We argue that the technical ‘friction’ inherent in AIGC functions as a subcultural filter; it transforms the audience from passive consumers into active ‘algorithmic critics.’ In these exclusive technical circles (Quanzi), identity is no longer awarded through shared feelings, but earned through the intellectual labor of deconstructing synthetic artifacts. This marks a fundamental shift from the affective resonance of the UGC era to the structural mastery of the AIGC era.

- It suggests that Friction shapes Identity.
- UGC often elicits “mindless positivity” (high sentiment, low friction). AIGC, being controversial and imperfect, elicits “mindful friction” (lower sentiment, high cognitive load).
- For Generation Z, identity is not just about what they *like*; it’s about what they *debate*. The controversy surrounding AIGC (Is it art? Is it ethical?) forces users to take a stand, defining their values more sharply than the passive consumption of UGC. Thus, the *process* of wrestling with the “fake” nature of AIGC ironically leads to a stronger (techno-centric) identity construction.

### Resolving the Simpson’s Paradox: a multi-group validation

A critical methodological imperative in cross-category modeling is ruling out Simpson’s Paradox, wherein a pooled-sample association might merely reflect a statistical artifact generated by distinct group mean differences. To rigorously test the validity of the negative sentiment-to-identity pathway, a Multi-Group Structural Equation Model (MG-SEM) was executed, specifying Content Type as the grouping constraint. The isolated group estimates fundamentally falsify the artifact hypothesis. Within the specific AIGC subsample, the structural path from Sentiment Score to Identity Reinforcement remained highly significant and negative ( β=−0.356,p<0.001 ). Similarly, the inverse structural relationship held within the UGC subsample ( β=−0.347,p<0.001 ). This structural consistency confirms that the “Authenticity Paradox” is not a mathematical illusion driven by spatial distances between content types. Rather, it operates as a genuine, endogenous behavioral mechanism: within the algorithmic media sphere, elevated interpersonal emotional warmth structurally suppresses subcultural identity formation, yielding the primary mediational space to cognitive mastery and rigid boundary-setting.

### AIGC’s “stealth” intervention in cultural production

As noted in the problem statement, AIGC intervenes “stealthily.” It does not scream for attention via emotion (where it fails); it seduces via *process* (Shneiderman, 2022). Users come for the spectacle, stay for the technical debate, and in doing so, are socialized into a “techno-identity.” This suggests that the future of cultural identity for Gen Z may be less about “shared values” and more about “shared tools.”

## Conclusion, limitations, and future perspectives

### Conclusion

This empirical study of 11,628 Douyin comments demonstrates that Generative AI (AIGC) and User-Generated Content (UGC) construct cultural identity through fundamentally different mechanisms.

1. UGC relies on an Affective Path: it leverages emotional authenticity and interpersonal connection to build broad, warm, human-centric identities.
2. Crucially, while our findings indicate AIGC tends to facilitate community formation through structural boundaries based on technical literacy rather than primarily through affective interpersonal bonds, this dynamic may not be static. As digital natives grow more accustomed to synthetic media, these initial technical barriers could ultimately evolve into new forms of social bonding.
3. The Parallel Mediation Model (Content → Tech & Circle → Identity) is a scientifically valid explanation for how AIGC influences Gen Z, highlighting that Technology Acceptance is the critical gateway for identity in the AI era (Mariani et al., 2023).

### Practical implications

- For platforms: To maximize user retention (Identity), platforms must balance the feed. Pure AIGC feeds risk “emotional burnout” (low sentiment). Algorithms should intersperse AIGC (for cognitive stimulation/circling) with UGC (for emotional grounding).
- For content creators: Human creators should not try to compete with AI on “perfection.” Their competitive advantage is “imperfection”—the flaw that signals humanity and triggers the “Warm Path” of identity.
- For youth education: Media literacy must expand to include “Algorithmic Identity (Ng et al., 2021)”. Gen Z needs to be aware of how their fascination with *tools* (AIGC) can subtly reshape their cultural values toward technocracy, potentially at the expense of humanistic empathy.

### Limitations

- First, the cross-sectional and observational nature of our dataset precludes firm causal interpretations. While Structural Equation Modeling (SEM) effectively delineates predictive and associative relationships between content types and identity reinforcement, the proposed dual-path mechanism remains a correlational framework. Longitudinal validations are required to establish definitive causality. Consequently, the structural pathways mapped within this dataset reflect predictive associations rather than deterministic causal trajectories. The shifting nature of youth subcultures and algorithmic platform designs means future experimental setups or multi-wave longitudinal panel data will be necessary to track the long-term causal evolution of this dual-path framework.
- Second, our dataset is derived exclusively from a single platform (Douyin). Although Douyin shares algorithmic similarities with global platforms like TikTok, regional nuances in digital socialization mean these findings might not perfectly generalize worldwide. Future research should test this mechanism across diverse global platforms to determine how the ‘authenticity paradox’ operates across cultural contexts.
- Methodology: The “Sentiment Score” is a proxy. It may not capture complex emotions like “irony” or “camp,” which are prevalent in Gen Z culture.
- Third, while the current dictionary-based structural equation modeling provides a robust foundational skeleton of the identity mechanism, it inevitably confronts the boundary conditions of linear additivity and static semantic extraction. Given the highly ephemeral and nuanced nature of Generation Z subcultural slang (e.g., irony, metaphor, and evolving algorithmic jargon), future research should transition toward Large Language Model (LLM)-assisted semantic extraction with human-in-the-loop validation to capture deeper contextual granularities. Furthermore, the interplay between technical jargon density, sentiment polarity, and identity formation may exhibit fundamentally non-linear topographies. Future studies are encouraged to deploy advanced machine learning algorithms (e.g., Random Forest or Gradient Boosting) to map these potential threshold effects. Most critically, the statistical pathways identified herein represent merely the surface of a deeper socio-technical infrastructure. The “Cool Path” is likely a dynamic, recursive loop where users’ technical engagement is continuously reinforced by both the platform’s algorithmic recommendation logic and the strategic intent of creators (e.g., deliberately embedding opaque model parameters to gatekeep communities). To fully capture this dynamic sufficiency, future investigations must employ Agent-Based Modeling (ABM) or system dynamics simulation. Grounding such computational simulations in the micro-level behavioral rules mapped in this study will help verify how these socio-technical interactions endogenously generate the macro-level “Authenticity Paradox” over time.

### Final word

Despite the technical novelty of AIGC, this study concludes that UGC possesses an irreplaceable core advantage in constructing profound and authentic cultural identity. AIGC is structurally associated with a “cool,” sharp, and stratified identity profile; UGC facilitates a “warm,” messy, and human identity. In the hybrid future of social media, the mental health and cultural cohesion of Generation Z will depend on maintaining the balance between these two forces.

## Data Availability

The datasets presented in this study can be found in online repositories. The names of the repository/repositories and accession number(s) can be found in the article/Supplementary material.
