# Supplementary material for: The mechanism of generative AI’s construction of cultural identity: an empirical study based on Generation Z’s social media behavior
Source: Front Psychol. 2026 Jul 17;17:1834334. doi: 10.3389/fpsyg.2026.1834334 (PMC13423852; doi:10.3389/fpsyg.2026.1834334)
Supplement: Supplementary file 1 [file Supplementary_file_1.DOCX]

**Supplementary Materials**

**Manuscript Title:** The mechanism of generative AI's construction of cultural identity: An empirical study based on Generation Z's social media behavior

**Appendix A: Exhaustive Technology Acceptance Lexicon** *Note: This custom dictionary captures the technical, algorithmic, and operational vernacular utilized by Generation Z on the Douyin platform. To ensure computational transparency and reproducibility, the core high-frequency terms utilized for the Net Technology Acceptance Score (Equation 1) are listed below.*

**Positive/Analytical Technical Terms (Original Chinese & English Translation):**

- **Core Algorithmic:** 算法 (Algorithm), 模型 (Model), 权重 (Weights), 参数 (Parameters), 训练 (Training), 采样 (Sampling), 迭代 (Iteration), 步数 (Steps).
- **Generative Operational:** 渲染 (Render), 提示词 (Prompt), 跑图 (Generation/Rendering), 微调 (Fine-tuning), 喂图 (Image-to-image/Feeding images), 垫图 (Base image generation).
- **Visual & Structural:** 分辨率 (Resolution), 引擎 (Engine), 构图 (Composition), 光影 (Lighting), 质感 (Texture), 骨骼绑定 (Rigging), 景深 (Depth of field), 细节 (Details).
- **Specific AI Frameworks (Jargon):** LoRA (Low-Rank Adaptation), ControlNet, Stable Diffusion, Midjourney, 虚拟人 (Virtual Human/Idol), AI绘图 (AI Painting).

**Negative/Skeptical Technical Terms (Original Chinese & English Translation):**

- **Algorithmic Artifacts:** 假 (Fake), 穿模 (Clipping/Glitch), 崩坏 (Distortion), 畸形 (Deformed), 多根手指 (Extra fingers), 电子包浆 (Digital artifact/patina).
- **Aesthetic Skepticism:** 恐怖谷 (Uncanny valley), 塑料感 (Plastic feel), AI味 (AI flavor/style), 拼凑 (Patchwork), 违和 (Incongruous), 生硬 (Stiff/Unnatural), 死板 (Rigid), 没灵魂 (Soulless).

**Appendix B: Exhaustive Sentiment Lexicon** *Note: This lexicon is adapted specifically for subcultural digital interactions on short-video platforms to calculate the Net Sentiment Score (Equation 3). It captures the nuanced emotional valences of digital natives, moving beyond standard generic dictionaries.*

**Positive Sentiment Terms (Affective Warmth & Praise):**

- **High Intensity:** 绝美 (Absolutely beautiful), 震撼 (Shocking/Mind-blowing), 惊艳 (Stunning), 绝了 (Amazing/Incredible), 牛逼 (Awesome/Epic).
- **Emotional Resonance:** 喜欢 (Like/Love), 治愈 (Healing), 感动 (Touched), 真实 (Real/Authentic), 温暖 (Warm), 爱了 (Love it), 共鸣 (Resonance).
- **Approval & Engagement:** 好看 (Good-looking), 完美 (Perfect), 期待 (Looking forward to), 舒适 (Comfortable), 厉害 (Impressive), 灵动 (Vivid/Dynamic), 支持 (Support), 宝藏 (Treasure).

**Negative Sentiment Terms (Critique & Alienation):**

- **High Intensity Disgust/Fear:** 恶心 (Disgusting), 害怕 (Scared), 吓人 (Terrifying), 阴间 (Creepy/Underworld), 诡异 (Weird/Eerie).
- **Quality & Rejection:** 劣质 (Low-quality), 离谱 (Outrageous), 讨厌 (Dislike), 失望 (Disappointed), 抵制 (Boycott), 反感 (Repulsed), 难看 (Ugly).
- **Emotional Detachment:** 冰冷 (Cold), 毫无意义 (Meaningless), 无语 (Speechless), 尴尬 (Awkward), 敷衍 (Perfunctory), 毁了 (Ruined).
